# Associations Between Subclinical Thyroid Dysfunction and Cardiovascular Risk Factors According to Age and Sex

**DOI:** 10.1210/clinem/dgae860

**Published:** 2024-12-12

**Authors:** Oliver Baretella, Manuel R Blum, Nazanin Abolhassani, Heba Alwan, Lea Wildisen, Cinzia Del Giovane, Kali Tal, Elisavet Moutzouri, Bjørn O Åsvold, Anne R Cappola, Jacobijn Gussekloo, Massimo Iacoviello, Giorgio Iervasi, Misa Imaizumi, Stefan Weiler, Salman Razvi, José A Sgarbi, Henry Völzke, Suzanne J Brown, John P Walsh, Bert Vaes, Bu B Yeap, Robin P F Dullaart, Stephan J L Bakker, Maryam Kavousi, Graziano Ceresini, Luigi Ferrucci, Drahomir Aujesky, Robin P Peeters, Douglas C Bauer, Martin Feller, Nicolas Rodondi

**Affiliations:** Institute of Primary Health Care (BIHAM), University of Bern, 3012 Bern, Switzerland; Department of General Internal Medicine, Inselspital, Bern University Hospital, 3010 Bern, Switzerland; Institute of Primary Health Care (BIHAM), University of Bern, 3012 Bern, Switzerland; Department of General Internal Medicine, Inselspital, Bern University Hospital, 3010 Bern, Switzerland; Institute of Primary Health Care (BIHAM), University of Bern, 3012 Bern, Switzerland; Department of Epidemiology and Health Systems, Center for Primary Care and Public Health (Unisanté), University of Lausanne, 1011 Lausanne, Switzerland; Institute of Primary Health Care (BIHAM), University of Bern, 3012 Bern, Switzerland; Institute of Primary Health Care (BIHAM), University of Bern, 3012 Bern, Switzerland; Institute of Primary Health Care (BIHAM), University of Bern, 3012 Bern, Switzerland; Institute of Primary Health Care (BIHAM), University of Bern, 3012 Bern, Switzerland; Institute of Primary Health Care (BIHAM), University of Bern, 3012 Bern, Switzerland; Department of General Internal Medicine, Inselspital, Bern University Hospital, 3010 Bern, Switzerland; HUNT Center for Molecular and Clinical Epidemiology, Department of Public Health and Nursing, NTNU, Norwegian University of Science and Technology, 7491 Trondheim, Norway; HUNT Research Centre, Department of Public Health and Nursing, NTNU, Norwegian University of Science and Technology, 7600 Levanger, Norway; Department of Endocrinology, Clinic of Medicine, St. Olavs Hospital, Trondheim University Hospital, 7030 Trondheim, Norway; Division of Endocrinology, Diabetes, and Metabolism, Department of Medicine, University of Pennsylvania School of Medicine, Philadelphia, PA 19104-5160, USA; LUMC Center for Medicine for older people, Department of Public Health and Primary Care, and Department of Internal Medicine, Section Gerontology and Geriatrics, Leiden University Medical Center, 2333 ZD Leiden, the Netherlands; Cardiology Unit, Emergency and Organ Transplantation Department, University of Bari, 70124 Bari, Italy; National Research Council, Institute of Clinical Physiology, Tuscany Region G. Monasterio Foundation, 56124 Pisa, Italy; Department of Clinical Studies, Radiation Effects Research Foundation, Nagasaki 850-0013, Japan; Department of Chemistry and Applied Biosciences, Institute of Pharmaceutical Sciences, ETH, 8093 Zurich, Switzerland; Institute of Primary Care, University and University Hospital Zurich, 8091 Zurich, Switzerland; Translational and Clinical Research Institute, Newcastle University, Newcastle upon Tyne NE1 3BZ, UK; Division of Endocrinology, Faculdade de Medicina de Marília, Marília, São Paulo 17519, Brazil; Institute for Community Medicine, SHIP/Clinical-Epidemiological Research, University Medicine Greifswald, 17489 Greifswald, Germany; Medical School, University of Western Australia, Crawley, Western Australia 6009, Australia; Department of Endocrinology and Diabetes, Sir Charles Gairdner Hospital, Nedlands, Western Australia 6009, Australia; Medical School, University of Western Australia, Crawley, Western Australia 6009, Australia; Department of Endocrinology and Diabetes, Sir Charles Gairdner Hospital, Nedlands, Western Australia 6009, Australia; Department of Public Health and Primary Care, KU Leuven, 3000 Leuven, Belgium; Medical School, University of Western Australia, Perth, Western Australia 6009, Australia; Department of Endocrinology and Diabetes, Fiona Stanley Hospital, Perth, Western Australia 6150, Australia; Department of Internal Medicine, University Medical Center Groningen, University of Groningen, 9713 AV Groningen, the Netherlands; Department of Internal Medicine, University Medical Center Groningen, University of Groningen, 9713 AV Groningen, the Netherlands; Department of Epidemiology, Erasmus MC, University Medical Center Rotterdam, 3015 GD Rotterdam, the Netherlands; Unit of Internal Medicine and Oncological Endocrinology, University of Parma, University Hospital Parma, 43121 Parma, Italy; National Institutes on Aging, Longitudinal Studies Section, Baltimore, MD 21225-1233, USA; Department of General Internal Medicine, Inselspital, Bern University Hospital, 3010 Bern, Switzerland; Department of Internal Medicine, Erasmus Medical Center, 3015 GD Rotterdam, the Netherlands; Institute of Primary Health Care (BIHAM), University of Bern, 3012 Bern, Switzerland; Department of Medicine, University of California, San Francisco, CA 94115, USA; Department of Epidemiology and Biostatistics, University of California, San Francisco, CA 94158, USA; Institute of Primary Health Care (BIHAM), University of Bern, 3012 Bern, Switzerland; Institute of Primary Health Care (BIHAM), University of Bern, 3012 Bern, Switzerland; Department of General Internal Medicine, Inselspital, Bern University Hospital, 3010 Bern, Switzerland

**Keywords:** dyslipidemia, high-sensitivity C-reactive protein, arterial hypertension, LDL-cholesterol, subclinical hyperthyroidism, subclinical hypothyroidism

## Abstract

**Context:**

Subclinical thyroid dysfunction (ScTD), comprising subclinical hypothyroidism (SHypo) and subclinical hyperthyroidism (SHyper), has been associated with increased risk for cardiovascular events.

**Objective:**

To assess associations between ScTD and cardiovascular risk factors (cvRFs) according to age and sex.

**Methods:**

This analysis of pooled participant data from large prospective cohort studies from the Thyroid Studies Collaboration assessed cvRFs (blood pressure [BP], lipid levels, high-sensitivity C-reactive protein [hs-CRP]) among participants aged 18 to 103 years with SHypo (thyroid-stimulating hormone [TSH] > 4.50 mU/L, normal fT4) and SHyper (TSH < 0.45 mU/L, normal fT4) vs euthyroid (TSH 0.45-4.50 mU/L).

**Results:**

Of 69 006 participants (mean age 62 years, 55% women, 25% current smokers) from 16 international cohorts, 3748 (5.4%) had SHypo and 3428 (5.0%) had SHyper. In both women and men, systolic and diastolic BP were similar regardless of thyroid status. Exceptions were lower diastolic BP in women with SHyper compared to euthyroid participants (adjusted mean difference [aMD] −1.3 mmHg, 95% CI −2.0 to −0.5), and lower systolic BP in men with SHyper compared to euthyroid participants (aMD −3.1 mmHg, 95% CI −4.8 to −1.4). In both women and men, lipid levels (total, HDL, LDL-cholesterol, triglycerides) and hs-CRP were similar regardless of thyroid status. The only exception were women with SHyper who had lower LDL-cholesterol vs euthyroid (aMD −0.17 mmol/L, 95% CI −0.29 to −0.05).

**Conclusion:**

Participants with ScTD and euthyroid participants have similar cvRFs and differences are arguably too small to explain the increased cardiovascular risk in ScTD observed in previous studies.

Subclinical thyroid dysfunction (ScTD) is defined by thyroid-stimulating hormone (TSH) levels outside and free thyroxine (fT4) concentrations within their respective reference ranges (1, 2); women are more likely to have ScTD—especially elevated TSH—than men (3-6). Both subclinical hypothyroidism (SHypo) and subclinical hyperthyroidism (SHyper) are linked to higher risk of coronary heart disease and associated mortality (2, 7-10). Although risk of ScTD and coronary heart disease both increase with age (3, 11, 12), the excess risk of coronary heart disease in those with ScTD does not increase with advancing age (8, 9).

SHypo has been associated with several modifiable cardiovascular risk factors, including hypertension (13, 14), obesity (15), and dyslipidemia (6), which may even extend to variations in the euthyroid range (16), but these associations have been inconsistent in studies using population-based data (17, 18). SHyper has not been associated with hypertension (13), but the underlying body of data is scarce. Overall, there is scarcity with regard to clinical trials on ScTD (19). Robust data on SHypo were published in an analysis focusing on the older population (20). SHypo is often treated with levothyroxine, but its effect on cardiovascular events and mortality is uncertain, since even the largest randomized controlled trial yet conducted on this topic was underpowered to assess cardiovascular events (19, 21, 22).

As there have been few large multinational analyses of the association between ScTD and cardiovascular risk factors, we worked within the Thyroid Studies Collaboration to pool individual participant data across large prospective cohort studies on multiple continents. To determine if this population with ScTD was at increased risk of cardiovascular events, we identified the cardiovascular risk factors most prevalent in these patients (8-10, 23, 24) and then determined whether these associations varied by age, sex, or TSH levels.

## Materials and Methods

### Cohort Data

We pooled individual participant data within the Thyroid Studies Collaboration (https://www.thyroid-studies.org) from all available cohort studies that: (i) measured baseline thyroid function (TSH, fT4); (ii) collected data on one or more baseline cardiovascular risk factors, such as systolic and diastolic blood pressure, circulating total cholesterol, high-density lipoprotein (HDL-), low-density lipoprotein (LDL-) cholesterol, triglycerides, high-sensitivity C-reactive protein (hs-CRP), and smoking status at baseline (25-27); and (iii) collected data on age and sex. We excluded participants taking the following outcome- or exposure-modifying medications: antihypertensive agents, lipid-lowering drugs, and exposure-modifying drugs such as thyroid hormones and antithyroid medication at baseline, and we excluded those for whom information on these medications was unavailable. We also excluded participants on antihypertensives from analyses of blood pressure and participants on lipid-lowering drugs from analyses of lipids and inflammation. For this reason, we excluded the HUNT study (28) from our analyses of lipid parameters as it collected no information on lipid-lowering medication.

### Cardiovascular Risk Factors

We evaluated baseline systolic and diastolic blood pressure, dyslipidemia with total cholesterol, HDL-cholesterol, triglycerides, and LDL-cholesterol. We used the Friedewald formula (29) to calculate LDL-cholesterol as appropriate (with values excluded if triglycerides > 4.52 mmol/L). We used baseline hs-CRP to identify low-grade systemic inflammation and excluded participants with values > 10 mg/L (30) as this may have signified the presence of an inflammatory process possibly due to an acute infection or critical illness which can alter thyroid function (31).

### ScTD and Subgroups

As for previous studies, we used uniform TSH cutoffs but cohort-specific fT4 cutoffs because of the poor harmonization of fT4 compared with TSH assays (8). We defined thyroid function categories as: (i) SHypo (TSH > 4.50 mU/L with fT4 within cohort-specific reference range; (ii) euthyroid state (TSH between 0.45 and 4.50 mU/L); and (iii) SHyper (TSH < 0.45 mU/L, fT4 within reference range). We excluded participants with overt thyroid dysfunction (TSH and fT4 outside the reference range) and with TSH values > 20 mU/L, but included participants with missing fT4 levels in the main analyses, since most people with normal TSH are euthyroid or can be categorized with subclinical rather than overt thyroid dysfunction (3, 8, 32). We excluded participants from the corresponding sensitivity analyses if their fT4 levels were missing. Additionally, a sensitivity analysis was conducted where analyses were limited to participants who have persistent SHypo and SHyper at follow-up. For this analysis, data on repeated thyroid function measurements and on thyroid-related medications were available in 4 cohorts (Bari, Busselton Health Study, Cardiovascular Health Study, Leiden 85+ Study) permitting corresponding analyses on persistent ScTD (33). In further subgroup analyses including all cohorts, we also compared participants with marked subclinical hyper- (TSH <0.10 mU/L) and marked SHypo (TSH 10-20 mU/L) to those in the euthyroid state (8), given that marked ScTD can be regarded as the most likely to persist as 50% of ScTD cases are reported to revert to the euthyroid state (34). We further analyzed subgroups by age (< 70 and ≥ 70 years old). Within each category, we calculated mean systolic and diastolic blood pressure (in mmHg), mean total, HDL-cholesterol and LDL-cholesterol, triglyceride levels (in mmol/L), and mean hs-CRP values (in mg/L).

### Statistical Analysis

To determine the association between ScTD (with euthyroid state as reference category) and each of the cardiovascular risk factors, we used univariate linear mixed-effects regression models. These models featured a random intercept for each cohort for continuous outcome variables in cardiovascular risk factors and mixed-effects logistic regression models, and a random intercept for each cohort for dichotomous outcome variables. For hs-CRP comparisons, we applied Poisson regression. We adjusted all analyses for age and current smoking status (25-27). Because outcomes differed by sex (highlighted by a significant *P* for interaction) and because of results of previous studies (35, 36), we stratified results for women and men separately. We explored subgroups by TSH categories for marked ScTD (< 0.10 and 10-20 with 0.45-4.50 mU/L as reference) (8), and by age strata < 70 and ≥ 70 years (37-39). This established age cutoff chosen provided similar numbers of participants for the examined cardiovascular risk factors. We repeated these calculations in corresponding sensitivity analyses without ScTD-classified participants when information on their fT4 levels was missing. In an additional analysis, we defined SHypo in participants ≥ 70 years using a cutoff for TSH > 7.5 mU/L (37). *P* values <.05 indicated statistically significant differences between groups. All analyses were performed with Stata version 15.1, Stata Corporation, Texas, USA.

## Results

### Cohort Characteristics

We analyzed data of individual participants that contained information on cardiovascular risk factors and corresponding medications from 69 006 participants in 16 cohorts in the Thyroid Studies Collaboration, spanning North America (USA), Europe (Belgium, Germany, Italy, The Netherlands, Norway, UK), and Australia (Supplementary Fig. S1) (40). Mean age was 62 (range, 18-103) years, 55% were women and 25% were current smokers (Table 1). Among participants, 5.0% had SHyper, and 5.4% had SHypo, including 0.9% with marked SHyper (TSH < 0.10 mU/L) and 0.6% with marked SHypo (TSH 10-20 mU/L). Medications to treat a thyroid disorder (thyroid hormones, antithyroid medication) were taken by 4.5%, 29% took antihypertensives, and 12% took lipid-lowering medication (Table 1). All treated participants were excluded in risk-specific analysis (eg, those on antihypertensive medication were excluded from the blood pressure analyses).

**Table 1.** Study characteristics

Study	N	Mean age (range)	Women (%)	Current smoking (%)	Subclinical hyperthyroidism		Subclinical hypothyroidism		Thyroid medication (%)	Antihypertensive medication (%)	Lipid-lowering medication (%)
					All (%)	Marked (%)	All (%)	Marked (%)			
HUNT	34 493	58 (19-98)	67	9715 (28)	1064 (3.1)	286 (0.8)	1291 (3.7)	129 (0.4)	1855 (5.4)	5526 (16)	N/A
PROSPER	4452	75 (70-83)	50	1175 (26)	117 (2.6)	29 (0.7)	285 (6.4)	22 (0.5)	0 (0)	3261 (73)	0 (0)
SHIP	4100	50 (20-81)	51	1314 (32)	1108 (27)	84 (2.0)	17 (0.4)	2 (0.0)	179 (4.4)	1202 (29)	314 (7.7)
HIMS	4041	77 (47-100)	0	194 (4.8)	49 (1.2)	8 (0.2)	296 (7.3)	22 (0.5)	120 (3.0)	2237 (55)	1533 (38)
CHS	3783	75 (64-98)	58	374 (9.9)	109 (2.9)	47 (1.2)	515 (14)	61 (1.6)	299 (7.9)	1951 (52)	184 (4.9)
HABC	2758	75 (69-81)	51	270 (9.8)	87 (3.2)	24 (0.9)	333 (12)	40 (1.5)	267 (9.7)	798 (29)	476 (17)
Whickham	2655	47 (18-93)	53	1258 (47)	265 (10)	0 (0.0)	310 (12)	34 (1.3)	78 (3.8)	169 (6.4)	17 (0.6)
Pisa	2632	61 (19-92)	31	1113 (42)	150 (5.7)	15 (0.6)	200 (7.6)	21 (0.8)	0 (0)	2049 (78)	N/A
PREVEND	2606	48 (28-75)	51	951 (36)	113 (4.3)	20 (0.8)	50 (1.9)	2 (0.1)	32 (1.2)	351 (13)	138 (5.3)
Busselton	2011	50 (18-90)	49	399 (20)	52 (2.6)	11 (0.5)	89 (4.4)	20 (1.0)	15 (0.8)	300 (15)	N/A
Rotterdam	1779	69 (55-93)	61	372 (21)	117 (6.6)	29 (1.6)	107 (6.0)	10 (0.6)	36 (2.0)	540 (30)	45 (2.5)
MrOS	1563	74 (65-99)	0	55 (3.5)	30 (1.9)	4 (0.3)	147 (9.4)	10 (0.6)	121 (7.7)	752 (48)	422 (27)
InChianti	1167	69 (21-103)	56	219 (19)	88 (7.5)	18 (1.5)	32 (2.7)	4 (0.3)	26 (2.2)	403 (35)	50 (4.3)
Leiden 85+	387	85 (85-85)	68	64 (17)	19 (4.9)	10 (2.6)	34 (8.8)	5 (1.3)	13 (3.4)	178 (46)	4 (1.0)
Bari	326	65 (22-93)	22	4 (1.2)	8 (2.5)	0 (0.0)	39 (12)	10 (3.1)	16 (4.9)	325 (99.7)	202 (62)
BELFRAIL	253	85 (80-100)	66	5 (2.0)	52 (21)	14 (5.5)	3 (1.2)	0 (0.0)	36 (14)	120 (47)	74 (29)
**Total**	**69 006**	**62 (18-103)**	**55**	**17 482 (25)**	**3428 (5.0)**	**599 (0.9)**	**3748 (5.4)**	**392 (0.6)**	**3093 (4.5)**	**20 162 (29)**	**3459 (12)**

Subclinical hyperthyroidism (TSH < 0.45 mU/L, normal fT4), marked TSH < 0.10 mU/L); subclinical hypothyroidism (TSH > 4.50 mU/L, fT4 normal), marked (TSH 10-20 mU/L); bottom line in bold with summarized numbers and values across all cohorts.

### Blood Pressure

**Figure 1.**
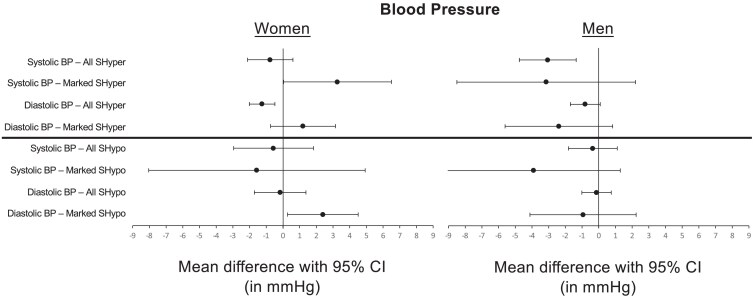
(16 Cohorts, *n =* 45 819 participants): mean differences of blood pressure in subclinical thyroid dysfunction *vs* the euthyroid state. Top: SHyper, subclinical hyperthyroidism (TSH < 0.45 mU/L, normal fT4) with marked (TSH < 0.10 mU/L) dysfunction; Bottom: SHypo, subclinical hypothyroidism (TSH > 4.50 mU/L, normal fT4) with marked (TSH 10-20 mU/L) dysfunction; mean differences with 95% CI vs the euthyroid state (TSH 0.45 to 4.50 mU/L) for systolic and diastolic blood pressure (BP) in mmHg for women (left) and men (right). Numerical details are displayed in Supplementary Tables S1A and S3A (40).

We did not report mean blood pressure values here, as they were unadjusted for age and smoking status, making a crude comparison between the thyroid categories (SHyper, euthyroid, SHypo) obsolete. In adjusted analyses, both women and men had similar systolic and diastolic blood pressure values across thyroid categories, with 2 exceptions: compared to euthyroid, women with SHyper had a lower diastolic blood pressure (adjusted mean difference [aMD] −1.3 mmHg, 95% CI −2.0 to −0.5), and men with SHyper had a lower systolic blood pressure (aMD −3.1 mmHg, 95% CI −4.8 to −1.4; Fig. 1, Supplementary Table S1A) (40). In the sensitivity analysis with persistent subclinical thyroid dysfunction, women with SHyper also had a lower diastolic blood pressure (aMD −10 mmHg, 95%CI −20 to −0.5; Supplementary Table S2A) (40). When we restricted the analyses to participants with marked SHyper (TSH < 0.1 mU/L) or marked SHypo (TSH ≥ 10 mU/L) and compared them to euthyroid participants, men had similar systolic and diastolic blood pressure values (Supplementary Table S3A) (40). However, women with marked SHyper had a higher systolic blood pressure compared to euthyroid participants (aMD 3.3 mmHg, 95% CI 0.0 to 6.5), and women with marked SHypo had a higher diastolic blood pressure (aMD 2.4 mmHg, 95% CI 0.3 to 4.5; Fig. 1, Supplementary Table S3A) (40). In sensitivity analyses excluding participants with missing fT4 values, results remained similar (Supplementary Tables S1C and S3C) (40).

### Lipids and Inflammation

**Figure 2.**
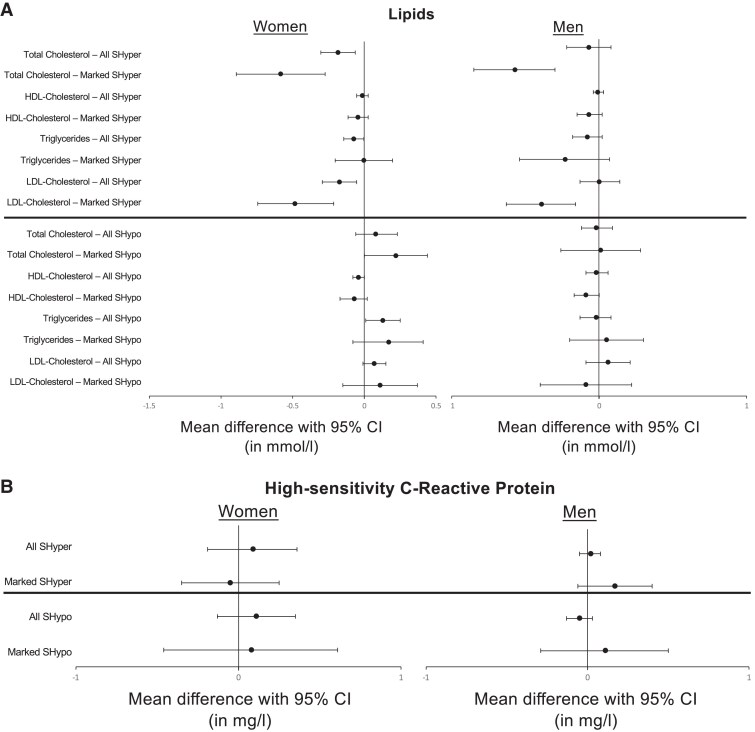
(13 Cohorts, *n* = 24 781 participants): mean differences of lipid parameters (top) and high-sensitivity C-reactive protein (bottom) in subclinical thyroid dysfunction vs the Euthyroid State. (A) Top: SHyper, subclinical hyperthyroidism (TSH <0.45 mU/L, fT4 within the reference range) with marked (TSH < 0.10 mU/L) dysfunction; Bottom: SHypo, subclinical hypothyroidism (TSH > 4.50 mU/L, fT4 within the reference range) with marked (TSH 10-20 mU/L) dysfunction; mean differences with 95% CI vs the euthyroid state (TSH 0.45 to 4.50 mU/L) for lipid parameters total cholesterol, high-density lipoprotein (HDL) cholesterol, triglycerides, and low-density lipoprotein (LDL) cholesterol in mmol/L for women (left) and men (right). Numerical details are displayed in Supplementary Tables S1B and S3B (40). (B) Top: SHyper, subclinical hyperthyroidism (TSH < 0.45 mU/L, fT4 within the reference range) with marked (TSH < 0.10 mU/L) dysfunction; Bottom: SHypo, subclinical hypothyroidism (TSH > 4.50 mU/L, fT4 within the reference range) with marked (TSH 10-20 mU/L) dysfunction; mean differences with 95% CI vs the euthyroid state (TSH 0.45 to 4.50 mU/L) for high-sensitivity C-reactive protein in mg/L for women (left) and men (right). Numerical details are displayed in Supplementary Tables S1B and S3B (40).

As with blood pressure, we did not report mean lipid or hs-CRP values here, as they were unadjusted for age and smoking status, making a crude comparison between the thyroid categories (SHyper, euthyroid, SHypo) obsolete. In adjusted analyses, both women and men had comparable lipid values across thyroid categories (Fig. 2, Supplementary Table S1B) (40). Of note, there were a few statistically significant differences in women (eg, total cholesterol between SHyper and euthyroid; HDL-cholesterol between SHypo and euthyroid; triglycerides between SHypo and euthyroid; Supplementary Table S1B) (40) that seemed clinically negligible. Statistical significance arose from the large number of study participants. The only exception were women with SHyper who had a lower LDL-cholesterol compared to euthyroid participants (aMD −0.17 mmol/L, 95%CI −0.29 to −0.05; Supplementary Table S1B) (40). These small differences were no longer statistically significant in the sensitivity analysis that only included individuals with persistent subclinical thyroid dysfunction at follow-up (Supplementary Table S2B) (40), possibly due to low statistical power. When we restricted the analyses to participants with marked SHyper (TSH < 0.1 mU/L) or marked SHypo (TSH ≥ 10 mU/L) and compared them to euthyroid participants, lipid values were again similar for both women and men across thyroid categories (Supplementary Table S3B) (40), with 2 exceptions: compared to euthyroid participants, women with marked SHyper had lower LDL values (−0.48 mmol/L, 95% CI −0.74 to −0.21), as did men with marked SHyper (−0.39 mmol/L, 95% CI −0.63 to −0.16; Supplementary Table S3B) (40). In sensitivity analyses excluding participants with missing fT4 values, results remained similar (Supplementary Tables S1D and S3D) (40).

Finally, we found no differences in hs-CRP levels between those with ScTD and euthyroid participants in either sex (Fig. 2, Supplementary Tables S1B, S2B, S3B, S1D, and S3D) (40).

### Subgroup Analysis by Age

Among male participants aged < 70 years, those with SHyper had a lower systolic and diastolic blood pressure compared to euthyroid participants (aMD −2.4 mmHg, 95% CI −4.2 to −0.7; and aMD −1.8 mmHg, 95% CI −3.0 to −0.7; Supplementary Table S4A) (40), which remained robust in sensitivity analyses excluding participants with missing fT4 values (Supplementary Table S4C) (40). Lipid values and hs-CRP were similar across TSH categories (Supplementary Table S4B) (40), also in sensitivity analyses excluding participants with missing fT4 values (Supplementary Table S4D) (40).

Among participants aged ≥ 70 years, there were no clinically meaningful differences in blood pressure across thyroid categories (Supplementary Table S5A) (40), which was substantiated in sensitivity analyses excluding participants with missing fT4 values (Supplementary Table S5C) (40). Similarly, there were no clinically meaningful differences in lipid values or hs-CRP across thyroid categories (Supplementary Table S5B) (40), congruent with sensitivity analyses excluding participants with missing fT4 values (Supplementary Table S5D) (40). After using a TSH cutoff of > 7.5 mU/L to define SHypo in the participants aged ≥ 70 years, clinically meaningful differences in blood pressure, lipid values and hs-CRP remained elusive (Supplementary Table S6A and S6B) (40).

## Discussion

This comprehensive analysis of data from 16 cohorts with 69 006 participants assessed associations between ScTD and cardiovascular risk factors to identify contributors to the observed increase in cardiovascular disease and mortality with ScTD (8, 9). Cardiovascular risk factors were similar between the ScTD and euthyroid groups; differences were of only marginal clinical relevance and irrespective of age and sex. Thus, classical cardiovascular risk factors do not appear to explain the increased cardiovascular risk in ScTD.

Men and women with marked SHyper (TSH < 0.10 mU/L) had consistently lower total and LDL-cholesterol levels than euthyroid individuals. Our results resemble those of a cross-sectional North American population-based analysis of individuals on outcome-modifying lipid-lowering drugs that found decreased lipid levels with low TSH levels (6). Individuals with lower TSH levels also had a more favorable lipid profile (lower total and LDL-cholesterol) in the Norwegian HUNT study (41), but we could not confirm the HUNT study's finding of a linear association between the entire TSH reference range and lipid parameters, as we had to exclude the HUNT cohort from the lipid analyses because information about lipid-lowering medication was not available.

In our study, participants with SHypo did not have an unfavorable lipid profile, contrary to the above-mentioned cross-sectional North American analysis on outcome-modifying lipid-lowering drugs, who reported increasing lipids with higher TSH levels (6). Unlike this study, ours excluded individuals on exposure-modifying thyroid medication and on lipid-lowering drugs from the corresponding lipid analysis, which may explain why our results differed. In the Norwegian HUNT study, as TSH increased within the reference range, it was associated with less favorable lipid concentrations (41). Yet again, since the HUNT study did not include information on lipid-lowering medications (41), we could not include this cohort in our corresponding analyses of thyroid function and lipids. A cross-sectional analysis of a general population in Denmark associated SHypo with increased triglycerides and low-grade inflammation in men (42). However, as the Danish study did not comment on lipid-lowering medication, we assume they did not exclude individuals on lipid-lowering drugs, which may have confounded CRP levels and contributed to differences between our results and theirs.

Women with marked SHyper had higher systolic blood pressure values in our study, which excluded those who had taken exposure- or outcome-modifying medication, as did the earlier meta-analysis that found increased blood pressure was associated with SHypo but not SHyper (13). However, we included mainly White individuals from eligible cohorts in the United States, Europe, and Australia, and we excluded the study from Japan because medication data were missing. Ethnic background and genetic differences may modify the association between SHypo and blood pressure and explain differences between our study and those that contained data from mainly Asian cohorts.

Although some previous studies suggested that ScTD was associated with increased cardiovascular risk (6, 13), our analysis of individual participant data did not show relevant associations between ScTD and the cardiovascular risk factors studied in this report. The statistically significant differences in the degree of cardiovascular risk factors were marginal from a clinical point of view, and most often in favor of ScTD and not the euthyroid state. Differences in LDL-cholesterol of 1 mmol/L are known to be clinically relevant as regards to cardiovascular risk (43). In our analyses, although statistically significant, mean LDL-cholesterol values differed by less than 0.2 mmol/L and this in favor of marked SHyper compared to the euthyroid state. Thus, our findings do not support the claim that those with ScTD are at higher cardiovascular risk due to higher blood pressure, dyslipidemia, or low-grade inflammation. These factors were regarded as possibly being responsible for a repeatedly observed higher risk of heart disease, cardiovascular events, and mortality in those with ScTD (8-10, 23, 24). In line with our present findings, a contemporary analysis did not reveal an association between subclinical thyroid dysfunction and incident diabetes even after adjustment for body mass index (33). One possibility would be that ScTD is an independent risk factor for cardiovascular disease (24). Several mechanisms have been suggested, including heart failure with prolonged cardiac isovolumic relaxation time, higher mean heart rate, and increased risk of atrial fibrillation, and the Thyroid Study Collaboration is investigating these (44).

### Strengths & Limitations

Our study was strengthened by a systematic review of all available population-based cohorts and our exclusion of participants who took exposure or outcome-modifying treatments, which might have biased previous analyses (6, 41, 42).

Our study was limited by the absence of data on specific medications for cardiovascular risk factors across cohorts, causing us to exclude participants from corresponding analyses. We had no information on menopause or postmenopausal hormone therapy, which may have influenced cardiovascular risk (45). We had no direct information regarding acute illnesses or recent radiologic studies with contrast agents that may have altered thyroid function in participants. However, we assume that such cofactors were rare in the individuals studied as we have excluded participants with hs-CRP levels > 10 mg/L from our analyses and data were mainly collected in an outpatient setting. Another limitation is that thyroid function was assessed at one timepoint; however, a sensitivity analysis including only individuals with persistent ScTD on follow-up showed similar results. Future studies should further investigate the causes of the association of ScTD with increased cardiovascular risk found in prospective observational studies (8). By using larger prospective datasets with data on thyroid function available at more than one time point, effects of persistent subclinical thyroid dysfunction may be assessed. Our data on participants with marked subclinical thyroid dysfunction could provide an outlook for such future analyses, since marked ScTD is the most likely one to persist.

Our large analysis of individual participant data from multiple cohorts found that ScTD and euthyroid have similar cardiovascular risk factors and differences are arguably too small to explain the increased cardiovascular risk in ScTD observed in previous studies.

## Data Availability

Restrictions apply to the availability of some or all data generated or analyzed during this study to preserve patient confidentiality or because they were used under license. The corresponding author will on request detail the restrictions and any conditions under which access to some data may be provided.

## Abbreviations

aMD: adjusted mean difference
cvRF: cardiovascular risk factor
fT4: free thyroxine
HDL: high-density lipoprotein
hs-CRP: high-sensitivity C-reactive protein
LDL: low-density lipoprotein
ScTD: subclinical thyroid dysfunction
SHyper: subclinical hyperthyroidism
SHypo: subclinical hypothyroidism
TSH: thyrotropin (thyroid stimulating hormone)
