# Correction to: “Associations Between Subclinical Thyroid Dysfunction and Cardiovascular Risk Factors According to Age and Sex”

**DOI:** 10.1210/clinem/dgaf061

**Published:** 2025-02-11

In the above-named article by Baretella O, Blum MR, Abolhassani N, Alwan H, Wildisen L, Del Giovane C, Tal K, Moutzouri E, Åsvold BO, Cappola AR, Gussekloo J, Iacoviello M, Iervasi G, Imaizumi M, Weiler S, Razvi S, Sgarbi JA, Völzke H, Brown SJ, Walsh JP, Vaes B, Yeap BB, Dullaart RPF, Bakker SJL, Kavousi M, Ceresini G, Ferrucci L, Aujesky D, Peeters RP, Bauer DC, Feller M, and Rodondi N on behalf of the Thyroid Studies Collaboration (*J Clin Endo Metab*. 2024; doi: 10.1210/clinem/dgae860), there was an error in the author notes and the Funding section.

In the originally published article, the author note “contributed equally” was attributed to authors Oliver Baretella, Manuel R. Blum, and Nicolas Rodondi. Martin Feller was excluded from this note, and the publisher acknowledges the error. In the corrected article, an asterisk signifying equal contribution has been added next to “Martin Feller” in the author list.

In the originally published article, in the Funding section, the first sentence read “This work was supported by the Swiss National Science Foundation (SNSF) grant number 32003B_200606 to (N.R.).” The parentheses around “N.R.” have been removed, so the sentence now reads “This work was supported by the Swiss National Science Foundation (SNSF) grant number 32003B_200606 to N.R.”

The article has been corrected online.

doi: 10.1210/clinem/dgae860

